# Anti‐ *Helicobacter pylori* Infection Treatment and Pulmonary Hypersensitivity: Case Series and Review of the Literature

**DOI:** 10.1111/crj.13816

**Published:** 2024-08-08

**Authors:** Shan Xu, Xiaohong Wu, Enguo Chen, Kejing Ying

**Affiliations:** ^1^ Respiratory and Critical Care Medicine, Regional Medical Center for National Institute of Respiratory Disease, Sir Run Run Shaw Hospital, School of Medicine Zhejiang University; ^2^ Cancer Center Zhejiang University Hangzhou China

**Keywords:** anti‐*H. pylori* infection treatment, bismuth‐containing quadruple therapy, furazolidone, interstitial lung disease, pulmonary hypersensitivity

## Abstract

**Background:**

*Helicobacter pylori* ( *H. pylori* ) infection is currently widespread throughout the world. Bismuth‐containing quadruple therapy is widely used, but it has rarely been associated with interstitial lung disease.

**Case Presentation:**

We described six cases with similar clinical symptoms and typical pulmonary interstitial imaging changes during anti‐ *H. pylori* therapy, usually on Days 7–12 following treatment. Anti‐*H. pylori* infection treatment was discontinued when it was suspected to be the cause of the clinical symptoms, and all of the patients accepted observation therapy. All of them had a favorable outcome, the clinical symptoms returned to normal almost 1 week later, and the chest computed tomography (CT) scan images showed remarkable absorption 4 weeks later.

**Conclusions:**

Drug interactions could be the cause, and the most likely drug was furazolidone. All of the patients recovered quickly after drug discontinuation, and low‐dose steroid may help shorten the recovery time.

## Graphical abstract

(A) Bilateral interstitial infiltrates in the lower lobe of case 1, (B) bilateral interstitial infiltrates in the lower lobe of case 2, (C) bilateral infiltration in the lower lobe of case 3, (D) bilateral little infiltration in the lower lobe of case 4, (E) bilateral infiltration in the lower lobe of case 5, and (F) bilateral interstitial infiltration and little pleural effusion of case 6 are shown.

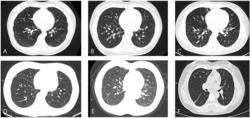

**Abbreviations**
CRP: C‐reactive protein
CT: computed tomography
HP:
*Helicobacter pylori*

PPI: proton pump inhibitor
WBC: white blood cell count

## 1 Introduction

*Helicobacter pylori* ( *H. pylori* ) is a gram‐negative spiral‐shaped bacterium that commonly colonizes the gastric mucosa of over half of the world's population [1]. *H. pylori* infection is typically acquired in childhood through oral‐oral or fecal‐oral transmission [2, 3] and can lead to various diseases such as gastritis, peptic ulcer, and gastric cancer [4, 5]. A standard triple therapy consisting of a proton pump inhibitor (PPI) and two antibiotics (clarithromycin and amoxicillin/metronidazole) is widely used as the first‐line regimen [6]. However, due to the increasing prevalence of *H. pylori* resistance to triple therapy, the eradication rate has been decreased [7]. Bismuth‐containing quadruple therapies, which include bismuth, a PPI, and two antibiotics such as amoxicillin and furazolidone, can achieve high eradication rates and are proposed to replace the standard triple therapy [8, 9]. Treatment regimens based on furazolidone have been reported in multiple studies to effectively eradicate *H. pylori* and are well‐tolerated by most patients [10, 11, 12]. However, pulmonary hypersensitivity can be found during treatment but is rarely reported. We described six cases with similar clinical symptoms and conducted literature review to better characterize this reaction.

## 2 Case Presentation

Six patients' information was reviewed (Table 1). The first three were hospital patients, and the other were outpatients, all of whom had a favorable outcome.

**TABLE 1:** Investigations, radiological changes, and treatment of the patients.

Variable	Patient 1	Patient 2	Patient 3	Patient 4	Patient 5	Patient 6
Outpatient/inpatient	In	In	In	Out	Out	Out
Age	45 y	36 y	35 y	34 y	57 y	52 y
Gender	Female	Female	Female	Female	Female	Female
Comorbidity	No	No	Rhinitis	No	No	No
Anti‐ *H. pylori* infection treatment	Rabeprazole; colloidal bismuth pectin capsules; amoxicillin capsules; furazolidone	Rabeprazole; colloidal bismuth pectin capsules; amoxicillin capsules; furazolidone	Pantoprazole; colloidal bismuth pectin capsules; amoxicillin capsules; furazolidone	Omeprazole; colloidal bismuth pectin capsules; amoxicillin capsules; furazolidone	Omeprazole; colloidal bismuth pectin capsules; amoxicillin capsules; furazolidone	Omeprazole; colloidal bismuth pectin capsules; amoxicillin capsules; furazolidone
	10 days	7 days	7 days	12 days	10 days	11 days
Fever	Yes	Yes	Yes	Yes	Yes	No
Cough	Yes	Yes	Yes	No	No	Yes
Muscle aches	Yes	No	Yes	No	No	No
Fatigue	No	No	No	Yes	Yes	No
Dyspnea	Yes	Yes	Yes	Yes	Yes	Yes
PaO2 (mmHg) (partial arterial pressure of oxygen)	70	82	88	90	75	73.5
Auscultation of lungs	Normal	Normal	Normal	Normal	Normal	Normal
Laboratory investigations						
WBC (10^9^/L)	10.2	4.1	11	10.1	9.2	15.8
Lymphocytes (10^9^/L)	1	0.5	1.32	0.64	1	0.61
Eosinophils (10^9^/L)		0.24	0.14	0.09	0.38	0.04
CRP (mg/L)	40.6	36.9	21.1	18.9	75.9	92.3
Sputum smear	Negative	Negative	Negative	Negative	Negative	Negative
Respiratory virus screening	Negative	Negative	Negative	Negative	Negative	Negative
CT scan	Bilateral interstitial infiltrates	Bilateral interstitial infiltrates	Bilateral infiltration and interstitial thickening	Bilateral infiltration and interstitial thickening	Bilateral interstitial infiltration	Bilateral interstitial infiltration and little pleural effusion
Treatment						
Antibiotic therapy	No	No	Ineffective	Ineffective	Ineffective	Ineffective
Hormonotherapy	Yes	No	No	No	No	No

### 2.1 Case 1

**FIGURE 1:**
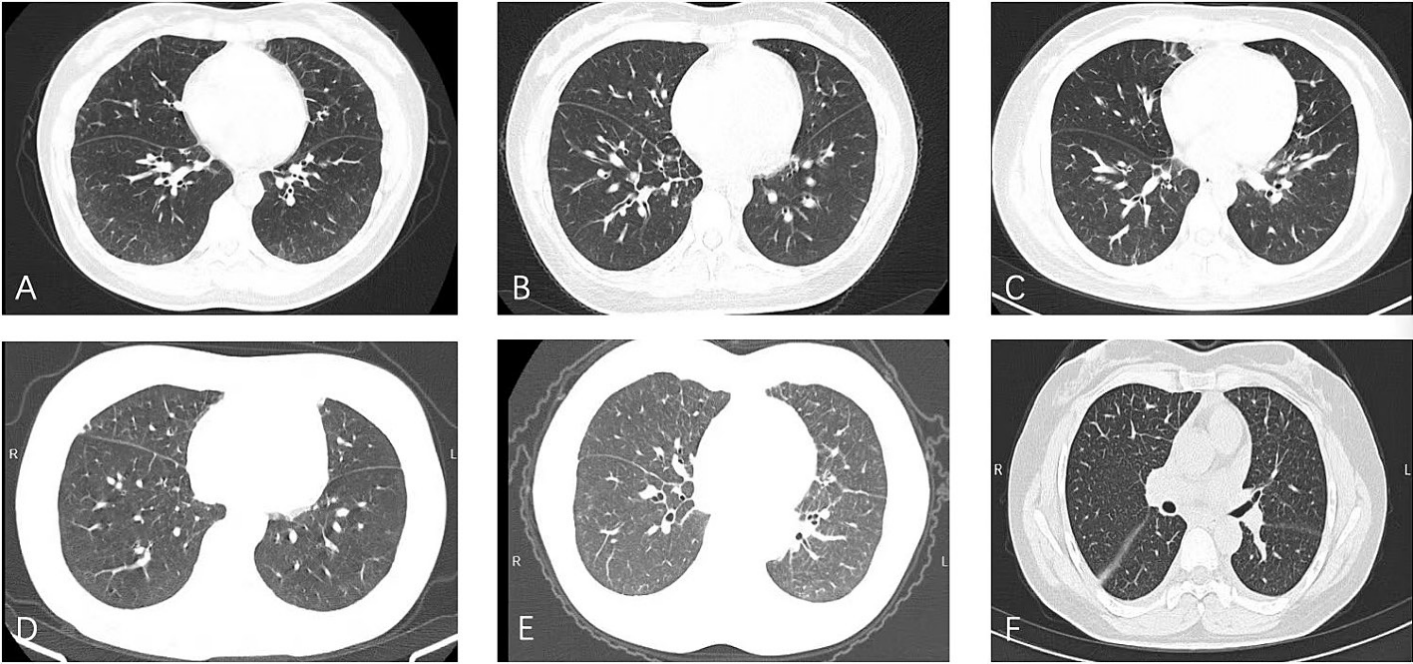
(A) Bilateral interstitial infiltrates in the lower lobe of Case 1; (B) bilateral interstitial infiltrates in the lower lobe of Case 2; (C) bilateral infiltration in the lower lobe of Case 3; (D) bilateral little infiltration in the lower lobe of Case 4; (E) bilateral infiltration in the lower lobe of Case 5; (F) bilateral interstitial infiltration and little pleural effusion of Case 6.

A 45‐year‐old female inpatient complained of a fever, nonproductive cough, muscle aches, and shortness of breath before being diagnosed with *H. pylori* infection and receiving quadruple therapy for 10 days. Auscultation revealed clear lungs, blood gas analysis indicated hypoxemia, and chest computed tomography (CT) scan images showed bilateral interstitial infiltrates, particularly in the lower lung fields (Figure 1A). The white blood cell count (WBC) of the patient was 10.2 × 10^9^/L, and the C‐reactive protein (CRP) level was 40.6 mg/L, but a sputum smear and respiratory virus screening were both negative. After ruling out respiratory tract infection, the most likely diagnosis was drug‐associated interstitial pneumonia, for which the patient was told to stop anti‐ *H. pylori* infection treatment and was treated with supplemental oxygen and oral glucocorticoid treatment (0.25 mg/kg). Her clinical symptoms returned to normal in 2 days, and her chest CT scan was close to normal after 4 weeks.

### 2.2 Case 2

A 36‐year‐old female inpatient complained of a fever, cough, and breathlessness before being diagnosed with *H. pylori* infection and received quadruple therapy for 1 week. Auscultation of the lungs was normal, but chest CT scan images showed bilateral interstitial infiltrates, particularly in the lower lung fields (Figure 1B). Her blood gas analysis was normal. Laboratory investigations showed lymphopenia, a CRP level of 36.9 mg/L, and negative sputum smear and respiratory virus screening. The patient was diagnosed with drug‐associated interstitial pneumonia, and she was given observation therapy while being told to stop anti‐ *H. pylori* infection treatment. Her clinical symptoms returned to normal 1 week later, and the chest CT scan was almost normal after 4 weeks.

### 2.3 Case 3

A 35‐year‐old female inpatient with seasonal allergic rhinitis complained of a fever, muscle aches, and minor shortness of breath before she was diagnosed with *H. pylori* infection and received quadruple therapy for 1 week. Lung auscultation was clear, and the blood gas analysis was normal. The chest CT scan images showed bilateral infiltration and interstitial thickening (Figure 1C). Despite mildly elevated CBC and CRP levels, the sputum smear and respiratory virus screening were negative. The initial diagnosis was respiratory tract infection, but the patient had a poor response to empiric antibiotic therapy before hospitalization. The patient's clinical symptoms improved until the anti‐ *H. pylori* infection treatment was stopped, and she finally was diagnosed with drug‐associated interstitial pneumonia and received observation therapy. Her clinical symptoms returned to normal 5 days later, and the chest CT scan was almost normal after 4 weeks.

### 2.4 Case 4

A 34‐year‐old female outpatient complained of a fever, fatigue, and a little shortness of breath. She was diagnosed with *H. pylori* infection and received quadruple therapy for 12 days. Lung auscultation and blood gas analysis were normal. The chest CT scan images showed little bilateral infiltration and interstitial thickening (Figure 1D). Laboratory investigations showed lymphopenia, a CRP level of 18.9 mg/L, and negative sputum smear and the respiratory virus screening. Although a respiratory tract infection was diagnosed during the early stage, the initial empiric antibiotic therapy was ineffective. The patient's clinical symptoms improved until the anti‐ *H. pylori* infection treatment was stopped, and she finally was diagnosed with drug‐associated interstitial pneumonia and received observation therapy. Her clinical symptoms gradually improved after 1 week, and the chest CT scan was almost normal after 4 weeks.

### 2.5 Case 5

A 57‐year‐old female outpatient complained of a fever for 1 day. She was diagnosed with *H. pylori* infection and received quadruple therapy for 10 days. Lung auscultation was clear, but the blood gas analysis showed hypoxemia. The chest CT scan images showed bilateral interstitial infiltration (Figure 1E). The WBC was normal, but the CRP level was 75.9 mg/L. Both the sputum smear and respiratory virus screening were negative. The initial diagnosis was respiratory tract infection, but empiric antibiotic therapy was ineffective during the early stage until the anti‐ *H. pylori* infection treatment stopped and the patient accepted the observation therapy. The patient's clinical symptoms gradually returned to normal 1 week later, and the drug‐associated interstitial pneumonia was finally diagnosed.

### 2.6 Case 6

A 52‐year‐old female outpatient complained of shortness of breath. She was diagnosed with *H. pylori* infection and received quadruple therapy for 11 days. The chest CT scan images showed bilateral interstitial infiltration and little pleural effusion (Figure 1F), the WBC was 15.8 × 10^9^/L, and the CRP level was 92.3 mg/L. The clinic diagnosis was respiratory tract infection, and empiric antibiotic therapy was given. The patient had a poor response to the initial therapy until the anti‐ *H. pylori* infection treatment stopped. Finally, she was diagnosed with drug‐associated interstitial pneumonia and accepted the observation therapy. Her clinical symptoms returned to normal 1 week later.

## 3 Discussion

*H. pylori* infection is extremely common worldwide. Because of the high prevalence of *H. pylori* infection and the implications for other diseases, *H. pylori* infection eradication treatment is crucial [13]. Combinations of bismuth, a PPI, and antibiotic agents have been commonly used as the first‐line therapy for *H. pylori* infection treatment, but the regimen associated with interstitial lung disease has been rarely reported. Here, we present six patients aged 34–57 years who developed pulmonary hypersensitivity as a result of anti‐ *H. pylori* infection treatment. In our cases, all the patients were females who complained of shortness of breath, and most of the patients presented with fever except one. Moreover, other clinical features similar to upper respiratory infection, such as cough, muscle aches, and fatigue, could be found in the medical history. Most of the patients developed clinical symptoms, usually on Days 7–12 following anti‐ *H. pylori* infection treatment, and similar chest CT scan images showed bilateral infiltration, interstitial thickening, and occasionally little pleural effusion. The laboratory investigations showed that CRP levels increased with a normal or slightly elevated leucocyte count, and common upper respiratory tract infection was ruled out in all of the patients with negative sputum smear results and respiratory virus screening. Four of these patients were initially treated with empiric antibiotic therapy, and all of them had a poor reaction. Anti‐ *H. pylori* infection treatment was discontinued when it was suspected to be the cause of the clinical symptoms, and all of the patients accepted observation therapy. One of them accepted small‐dose corticosteroid therapy (0.25 mg/d) for a short time because of obvious hypoxemia and chest CT scan imaging features. All of them had a favorable outcome. The clinical symptoms returned to normal almost 1 week later, and the chest CT scan images showed remarkable absorption 4 weeks later.

Upon review of the quadruple anti‐ *H. pylori* treatment regimen, it was determined that all of them accepted colloidal bismuth pectin capsules, amoxicillin capsules, and furazolidone. Two of the patients were treated with rabeprazole, one was treated with pantoprazole, and the others were treated with omeprazole. Among these drugs, furazolidone has been most commonly associated with pulmonary allergy, despite its excellent eradication rate in treating *H. pylori* [ 12, 14]. Furazolidone, a bactericidal agent, has a similar chemical structure to nitrofurantoin, which may cause pulmonary infiltration due to a hypersensitivity reaction [15]. The typical clinical manifestations in the acute form include cough, dyspnea, and fever often accompanied by muscle aches; in addition, diffuse interstitial infiltrates could be seen on chest CT scan. Among other drugs, rabeprazole as a PPI has ever been reported to cause interstitial pneumonia, with a good prognosis [16]. No association with pulmonary hypersensitivity has been reported for colloidal bismuth pectin capsules, amoxicillin capsules, and the other two PPI drugs.

In this report, furazolidone was given to all the cases presented here, one‐third of patients received rabeprazole, all of the patients developed pulmonary hypersensitivity, and clinical symptoms appeared on Days 7–12 after anti‐ *H. pylori* infection treatment, which was longer than previously reported following a 4‐ to 5‐day course of furazolidone [17, 18]. Most of the patients presented with symptoms similar to the reaction of those receiving furazolidone treatment, such as shortness of breath, fever, cough, and similar radiographic findings, such as bilateral interstitial infiltration. Furthermore, we suspected that the symptoms were caused by drug interactions, given the negative results of the sputum smear and respiratory virus screening, as well as the poor response to empiric antibiotic therapy. The most likely drug was furazolidone, and not rabeprazole or anything else.

We searched relevant literature reports, among which a total of five cases of pulmonary hypersensitivity caused by furazolidone were reported, three cases caused by taking furazolidone alone, including two cases of male and one case of female, and the remaining two cases of pulmonary hypersensitivity caused by *H. pylori* infection treated with a quadruplex bactericidal regimen containing furazolidone, both of which were female [14, 17, 18, 19]. In this paper, we reported six patients who also developed pulmonary hypersensitivity due to treatment against *H. pylori* infection, and all of the patients were female, which was consistent with the gender reported earlier, suggesting that female patients should be more vigilant about pulmonary hypersensitivity in the process of anti‐ *H. pylori* infection with the quadruplex bactericidal regimen containing furazolidone.

In general, patients exhibit prompt recovery after drug discontinuation, but corticosteroid therapy occasionally needs nitrofurantoin‐induced acute, subacute, and chronic pulmonary reactions [10, 14, 15, 20]. To date, there is no standard treatment, only some case reports. In our cases, all the patients stopped the anti‐ *H. pylori* infection treatment once the drug interaction was considered, and one patient who accepted steroid therapy for a short time had a quick recovery in 2 days, while the other patients recovered in 5–7 days. All the patients were advised to delay anti‐ *H. pylori* infection treatment, and empiric furazolidone should be avoided as much as possible in subsequent treatment.

## 4 Conclusion

Eradication of *H. pylori* infection is important. To date, bismuth‐containing quadruple therapies have been widely used, with furazolidone as an antibiotic. Currently, the anti‐ *H. pylori* infection regimen related to interstitial lung disease has received little attention. We reported six cases with similar clinical symptoms, usually on Days 7–12 following anti‐ *H. pylori* infection treatment. In our paper, they are all females, and they always presented with cough, fever, breathlessness, muscle aches, and fatigue, with bilateral infiltration on chest CT scans, which was similar to nitrofurantoin‐induced pulmonary hypersensitivity. After drug discontinuation, the patients recovered quickly in 5–7 days, and small‐dose corticosteroid therapy (0.25 mg/d) for a short time may shorten the recovery period but needs to be tested in large clinical trials in the future.

## Author Contributions

S.X. contributed to the study design and drafted the study. X.H.W. wrote the article. K.J.Y. and E.G.C. performed data analysis and gave the final approval of the version to be submitted. All authors read and approved the manuscript.

## Ethics Statement

This study was conducted in accordance with the Helsinki Declaration II and was approved by the Institutional Review Boards of Sir Run Run Shaw Hospital, School of Medicine, Zhejiang University.

## Consent

Written informed consent was obtained from individual or guardian participants. All six patients provided written consent to use their data for research purposes, and specifically, for publication of this manuscript.

## Conflicts of Interest

The authors declare no conflicts of interest.

## Data Availability

The data that support the findings of this study are available from the corresponding author upon reasonable request.
